# Abstracts and Gleanings

**Published:** 1882-03-20

**Authors:** 


### ABSTRACTS AND GLEANINGS.

**Asiatic Cholera and Yellow Fever—Experience in the United States Respecting Them.**—Dr. J. S. Billings, in American Journal of Medical Sciences, October, 1881, gives the following:

I. As regards cholera, this experience accords in most points with the conclusions of the Conference of Constantinople and Vienna. The quarantines of the United States have not been, and are not now, capable of preventing the importation of cholera. In the United States there is special danger of such importation through the personal baggage of emigrants.

When the disease has been introduced, more can be done in stamping it out, and in the way of personal prophylaxis, than in other pestilential diseases. The systematic disinfection of excreta, clothing, and persons, and the securing pure food and drink, are the means to this end.

II. As regards yellow fever, the epidemics of 1878 and '79 have produced considerable change in the opinions of American physicians. The majority believe that it is a specific disease—not connected with marsh malaria—due to a specific living cause, and not indigenous to, or endemic in, the United States, in which country it is always due to importation.

III. This importation might, to a great extent, but not entirely, be prevented by a proper system of quarantine, without causing undue obstruction to travel or traffic. The word quarantine includes what the Vienna Conference terms systems of medical inspection, either of ships, persons, or cargo, beyond the time needed for cleansing and disinfection.

IV. Measures which, without entirely preventing, will delay the importation of yellow fever, have a much greater relative importance than in other diseases, and for this reason inland quarantines are of much more value in this disease than in cholera.

V. The experience of 1879 is favorable to attempts to stamp out yellow fever by systematic cleansing and disinfection of the infected localities, and also by measures of depopulation and the formation of camps.

VI. Much of the practical difficulty in dealing efficiently with yellow fever being connected with diagnosis, attention is called to a recent attempt to formulate a diagnosis of this disease for sanitary purposes.

VII. From the practical as well as the scientific point of view, the great disideratum at present, as regards both cholera and yellow fever, is a test for the presence of their causes, other than the production of the disease in man.

VIII. Yellow fever cities are filthy cities, and yellow fever ships are usually foul ships. But we know of no city in the yellow fever region that is clean enough to make this rule of much importance as regards them, and it would be very unsafe to act on the supposition that a clean ship cannot become infected.

IX. The greatest difficulties in the way of preventing the introduction of cholera and yellow fever into the United States, are due to want of reliable information as to the sanitary condition of foreign ports, and of vessels sailing therefrom. To overeome these difficulties, co-operation between the great commercial nations of the earth is desirable.

X. Some account is given of the effort of the National Board of Health to secure this co-operation through the International Sanitary Conference of Washington, and of the results of this Conference. The conclusions of this Conference are not satisfactory, and will give no practical result. The proper basis for an international agreement on the subject is the following:

1. Each government should secure prompt and reliable information as to the existence of cholera, yellow fever, and plague, within its boundaries, and especially in its seaports.

2. Each government should communicate this information to the other parties to the agreement, and especially to their consuls, or consular agents, at the sea-port. In case of the occurrence of either of the above named diseases, the communication should be made with the least possible delay—by telegraph, if practicable.

3. When bills of health are required by any one of the contracting governments, such bills should be signed by its own agents at the port of departure, and these agents should have the right to make such inspection of the ships as is necessary to enable them to certify to the bill of health.

4. The agents charged with the duty of signing the bill of health, should also have the privilege, in case either cholera, yellow fever, or plague exists at the port of departure, of making use of the telegraph to notify their respective governments of the departure of ships from such infected ports.

5. The bill of health should be of the form approved by the Washington International Sanitary Conference—it should be neither a clean bill, nor a foul bill, but a certificate in detail as to the sanitary condition of the port, and of the ship.

**Blood-letting.**—Dr. Mays, in a paper before the San Francisco Medical Society, (Western Lancet) very sensibly remarks:

Were our grandfathers right or wrong? Was bloodletting founded on principles so fallacious as to warrant its total extinction? Without entering upon a question of so great magnitude just now, I will venture to say that none of the great modes of thought that have ever dominated medicine are really dead. Methods of treatment change, but the principles of our science remain about the same they ever were. What medical man rejects the doctrine of fluxion, or refuses to believe that in acute inflammation the current of blood setting towards the irritated organ may be turned aside by venesection, and the progress of the disease stayed thereby?

The medicine of to-day is not built upon the ruins of the medicine of yesterday; it may rather be likened to the upper tier of bricks in a noble though unfinished edifice. Yet there are many who imagine that the new must necessarily be a reversal of the old. As an instance of the current adoration of the brand-new, think how that useful, if a trifle pompous, word *antiphlogistic* has been dropped entirely out of our vocabulary. The medical writer of the day would consider himself disgraced if he used it He disowns both the word and the ideas it represents; yet at the next bedside he visits he puts into practice the very theory he derides. He does not fail to place his fever patient under regulations as to a low and carefully restricted diet—in other words, he carries out the antiphlogistic regimen. This is simply medical vandalism.

There is no danger of a revival of bleeding in its old aspects, nor do I wish to be understood as favoring such a movement; my plea is only for a more considerate bearing towards the forms of thought, our direct progenitors in the line of medical evolution. I would like to see the younger generation of physicians neither oblivious to the fact that venesection is often the very best therapeutic measure at their command, nor afraid to resort to it if they wish to.

**The American Public Health Association.**—The New York Medical Times (Homœopathic) thus speaks of the Public Health Association at Savannah :

All schools of medicine meet on the common ground of sanitation, or care of the public health. On this occasion, at Savannah, there was noticeable the pleasant mingling of the blue and the gray—the old Surgeon-General of the Confederate army (S. P. Moore), Majors, Captains, etc., of the same, cordially greeting their once Northern enemies; and the fraternizing of members of the two schools of medicine, which was largely promoted by our Dr. Falligant, who gave the only reception to the members of the A. M. H. Association. The generous hospitality of the Doctor and Mrs. F. was met by a pretty general attendance of all visiting members of the Association, and therapeutics found no place in the discussion of the excellent viands. The other social feature of the occasion was the excursion down the river to Tybee Island and return, given by the citizens of Savannah. A delightful *summer* afternoon, a bountiful collation with champagne, music and the Savannah ladies, made the affair a grand success.

The sessions of the Association were well attended by members, but not by citizens, except in the evenings. Discussions on papers read, showed a pretty thorough knowledge of the subjects under consideration, but there was the usual amount of time wasted on constitutional questions and resolutions which ought to have been tabled at once. One of these is worth noting here, for if its instructions were acted upon, the Homœopathic school would be left without representation in the National Board of Health. The resolution was offered on the false supposition that this Board would soon expire under the act organizing it; and provided that said Board be hereafter composed of members selected from the army and navy surgeons and members of State Boards of Health.

Such a formation of the National Board would effectually prevent popular representation on it. But after a long and warm discussion, a vote was taken which dropped the matter for the present.

**Small-Pox.**—The period of incubation of vaccina is from seven to nine days, so it is of the highest importance to vaccinnate as soon as the person is exposed, thereby superseding small-pox three days; or, if it must come, rendering it lighter. I am well aware that the adherents of vaccination are growing less year after year, but as it is the best preventive I know of, I shall continue to advocate it until something better is discovered. History tells us that before Jenner's time, it was estimated that there were about 600,000 deaths in the world, yearly, from this disease, and that Europe alone lost about 300,000 annually. I think it hardly reaches that at the present day, and as for its inducing other diseases, we have not sufficient grounds to rely upon.

Generally, the first symptom of small-pox is a decided chill or a chilly sensation, lasting a short time, followed by a fever, quite intense, on the tenth day after the exposure. This continues for a day or two with severe pain in the back, then small red spots will make their appearance, usually on the face first, having a granulated feel under the skin. The same manifestations may be seen in the throat and velum. After a few hours a clear fluid makes its appearance in these papules, converting them into vesicles, and soon a small, black depression may be seen on the top of the vesicle, called the umbilical spot, probably so called because it does not look like an umbilicus. This is the diagnostic mark where the other symptoms prevail. Chicken pox, or varicella, has the same general history. It is not my purpose, in this article, to enter into any detailed description of this disease, for that can be found in almost every text-book; nor to spend much time on the treatment, as different schools of medicine have different ideas in relation to it. But certainly, a reduction of the fever is of great importance, and I know of nothing better than aconite, gelseminum or veratum viride, to accomplish this end. The fever usually subsides spontaneously after the eruption fully appears, to re-appear again when the pustula stage commences. This may be due to the increased inflammation or from the formation of pus, or possibly to pyema. Tartar emetic in about one-hundredth grain doses is said to work wonders in mitigating the severity of the disease, and some have gone so far as to declare that it aborts it. Tartrate of potassa, dissolved in water, sufficiently weak to be made palatable for a drink, works magically in some cases; but, as a general rule, the disease will run its course, and a good treatment is to let it alone, lest harm be done. Give remedies to mitigate the great suffering as much as possible. Some mild narcotic or anodyne with pure, fresh air, frequent sponging of the surface with weak vinegar and water, or a solution of carbonate of soda, protecting the face from the air and light as much as possible, to prevent pitting, is good treatment and will carry more through safely to recovery than too much medication. And pray God that you do not catch it yourself. It is not considered contagious in its incipient stages, or when first recognized by a careful physician, but highly in the pustular and dessicating stages. The scabs should all fall off, and the body should be thorougly cleansed, before the patient is allowed to mingle with the outer world.

About ten drachms of nitrate of lead dissolved in a gallon of pure water, to which add a tablespoonful of salt, will make one of the best disinfectants that can be used. Sprinkle the bed with it, keep clothes wet with it hung all over the room, and use it freely in the chamber utensils.

Heat is the best known disinfectant for clothing—the hotter the better—a blaze is the best.—*Investigator.*

**The Period of Gestation and the Placenta of the Elephant.**—The birth of a young elephant, at Bridgeport, is the second well authenticated instance of such an occurrence in captivity on record, a previous birth having taken place in Philadelphia in March, 1880.

Professor Owen, of London, in Vol. III, page 742, of his Anatomy of Vertebrates, mentions still another instance as having occurred August 3, 1865, the elephants having paired December 18, 1863, but does not state where the birth took place, nor does he cite any authority in reference to it.

Professor H. C. Chapman, of Philadelphia, who, from his connection with the Zoological Garden there, and his interest in comparative anatomy, had an opportunity to follow events in the case of the elephant Hebe from the first suspicions of pregnancy to parturition, made an interesting communication to the Academy of Natural Sciences, of Philadelphia, to be found in Vol. VIII., of its Journal, upon the placenta and generative apparatus of the elephant, presenting at the same time the injected placenta.

In the case of Hebe the first coitus took place May 29th, and the last June 20th, 1878; eight months later, there being good reasons to consider her pregnant, the question of the period of gestation arose, in regard to which we quote from Dr. Chapman's paper :—

"Here again I was comparatively in the dark. In the Thesaurus of Seba, published in 1734, there is figured the fœtus of an elephant without any membranes, taken out of its mother at about the middle of the period of gestation. Zimmermann also gives a figure of a fœtus. In the description of this fœtus only vague allusions are made to the length of gestation. As is well known, among the ancients, Pliny thought the period of gestation was six months, Strabo about eighteen; according to Aristotle, however, nearly two years.

"What I had learned from travelers in the East, and from the case referred to by Professor Owen, the time being, in that instance, five hundred and ninety-three days, together with the fact of Aris'otle giving nearly two years, led me to indicate that about the first of March, 1880, would be the time at which the birth of the elephant might be looked for. The young elephant was born March 9th, exactly twenty months and twenty days after the last copulation, or twenty-one months and fifteen days, reckoning from the first one. The fixing of the period of gestation in the elephant at six hundred and thirty to six hundred and fifty-six days, is another interesting illustration of modern investigation confirming the statements made by that most profound thinker and careful observer, Aristotle.

"The labor was a very short one, the mother standing on all-fours, with one hind foot slightly raised. The head presented. The umbilical cord broke, and was removed with the placenta and membranes shortly afterward by Mr. Arstingstall. Immediately after birth the mother rolled the young one in the straw. The young elephant, a female, stood thirty inches in height, measured from base of trunk to root of tail, thirty-five inches, and weighed two hundred and thirteen and one-half pounds. It was noticed immediately that it sucked with the mouth, and not with the trunk, as Buffon reasoned it must do. * * * * *.

"The placenta of the elephant is not only interesting on account of its rarity, but also from its combining the characters of the placentæ of three different sets of animals. The impossibility of using the placenta, in the case of the elephaut at least, as a means of classification is therefore sufficiently obvious."

The latest elephantine baby is stated to have weighed one hundred and forty-six pounds at birth, and the period of gestation, as given, was shorter by several weeks than in the case reported by Dr. Chapman.—*Boston Med. and Surg. Journal.*

**Curability and Treatment of Pulmonary Phthisis.**—Before having proved anatomically (as has been done recently) that tubercle had a natural tendency to heal, the curability of pulmonary phthisis was a clinical fact well demonstrated, and which M. Jacquoud had contributed both in his teachings and in his writings. His new book dedicated to the curability of consumption is a new affirmation, more complete and more decisive, to practitioners, and ought to be their constant study. This book furnishes details very interesting of the greatest value.

In the use of cod liver oil M. Jacquoud protests energetically against the insufficiency of the doses usually given. Six teaspoonfuls is the minimum dose given by Dr. J. Occasionally larger doses are given, commencing with small and gradually increasing each day so as to reach each week an additlonal spoonful. Various means are used to produce tolerance of the oil. Alcohol is the best; strychnine and ether are also good.

Fever alone (by reason of the alteration produced in gastric secretion), is a contra-indication to cod liver oil, and then glycerine should be well digested during the fever. As to the hot season, it will be no obstacle to giving the oil. Glycerine, though inferior to cod liver oil, is a good addition, and given in from 40 to 60 grammes daily, is only contra-indicated in persons of cerebro-cardiac excitability, in insomnia, and in high temperature. In addition, give one drop of the essence of mentha, 10 grammes of cognac or rum, which makes the mixture more agreeable.

Arsenic is also a remedy which Dr. J. associates with those above mentioned, and is contra-indicated when there is lassitude after a walk, and the medicine, if continued, causes feebleness in the lower extremities. This is often the first symptom of arsenical saturation.

Among other remedies, creasote more rapidly and more surely diminishes expectoration and lessens bronchial lesions. This remedy unhappily produces certain difficulties. Commence with small doses, say from 3 to 5 grains, and gradually increase. It is better to give it in capsules, or to add to the oil or glycerine containing it, a drop of mentha.

The indications for the treatment of fever are variable. Quinine and especially the bromo-hydrate of quinine, is preferred, especially when there are cavernous indications or putrid expectoration, and then salicylic acid is also appropriate. The first day 30 grains of salicylic acid is given, and one-half this quantity each succeeding day. If the fever do not moderate, the dose is continued at 30 grains, in combination with cognac or rum, followed by a glass of water. If there be gastric intolerance, use the salicylate of soda, from 4 to 6 grammes, or with small doses subcutaneously. D. Jacquoud has studied the employment of benzoate of soda, by inhalations, so much praised by some physicians, but his conclusions are not so favorable as to lead him to continue it.

Dr. J. has seen all the localities he recommends, for phthsical patients and he favors the high latitudes of Davos de Saint Moritz et de la Maute Engadine.—*Journal de Therapəutique.*—*Therapeutic Gazette.*

**Small-Pox and Vaccination.**—M. Greenwood reports some interesting cases, which occurred in a recent epidemic of small-pox, and which illustrate very forcibly the protective effect of vaccination.

1. A woman suffering from small-pox suckled her child throughout nearly the whole period of the disease. The latter, vaccinated successfully a month before, never showed any symptoms.

2. Small-pox occurred in a German family and the oldest of four children eventually succumbed to it He was the only one in the family unvaccinated, and was nursed for nearly a week at home among the other three, all of whom escaped. The father believed in vaccination, but living in the United States at the time when this child was born, and vaccination being non-compulsory, he had neglected to have it done.

3. Small-pox broke out in a family of three children, none vaccinated. On the removal of the oldest, aged seven, to the hospital, the other two were vaccinated. though the mother said that the second child, aged five years, was not feeling very well at the time. The vaccination was successful in both cases; but in that child, as the vaccinal pustules were beginning to die away, just ten days after vaccination, a slight but distinct variolous eruption broke out over the body. The unvaccinated child died of the disease; the other scarcely had any constitutional symptoms at all, the vaccine, owing to its shorter period of incubation and action, having anticipated the action of the variolous poison, which was already in the system.—*British Med. Journal.*

**The Use of Boracic Acid and Calendula in Ear Diseases.**—Dr. Sexton in the Medical Society of New York (Med. Record) admits a boracic acid mixture as follows: This mixture is made of equal parts by weight of tincture of calendula and finely powdered boracic acid, as follows: Evaporate the calendula down in a water-bath, at a temperature of 150° F., to a pasty consistency, and then mix with one-half of the boracic acid; evaporate to dryness, add the other half, and triturate. I do not always employ this preparation in its full strength of the calendula, but frequentiy find it sufficiently strong when reduced by the addition of more or less pulverized boracic acid.

The local action of both of these remedies on supporating surfaces seems to be somewhat similar; they are, perhaps, antiseptic, and it is their nature to promote healing; by means of their action secretions are checked, odors are stopped, and healthy granulation promoted. In some otorrhœas the boracic acid may be employed as a dressing, and will not be found so heating as cotton-wool· In this way a light crust is sometimes formed over ulcerating or secreting surfaces, protecting the former while cicatrization goes on, and preventing the latter, when retained, from macerating the neighboring parts and undergoing decomposition

In by far the greater number of cases of suppurative otitis media, I prefer these drugs in combination. Their range of application is thus very extensive; when used in traumatic rupture of the drum-head, and in the myringitis accompanying perforative inflammation of the middle ear, the results have been most gratifying. In ulcerative inflammation of the external auditory canal of children, especially in the neglected cases among the poor, where the discharge is sanguineous, purulent, and highly offensive, the mixture is very efficacious.

After the removal of polypi, as soon as the bleeding has been arrested, it is sometimes useful.

The powder may be introduced into the ear through an aural speculum, or it may be blown in with a small insufflator.

In acute cases, the powder should at first be applied with great care, for in some instances it is not well borne at the beginning of the attack.

In nearly all cases, the discharge may be expected to lessen under this treatment; and when odor is present, its disappearance may be confidently expected to take place very soon.

**The Treatment of Sea-Sickness.**—Dr. Milan Soule, surgeon on the steamship City of Sidney, has written an account of his experience with the bromide treatment for sea-sickness, as laid down by Dr. G. M. Beard. His testimony to its efficacy is very emphatic and convincing. He says:

"About three years ago I began to use the bromides in treating sea-sickness, following, as nearly as possible, the direction given in Dr. Beard's valuable monograph on that subject. I had then been in the service of the Pacific Mail Steamship Company nearly four years, and as my field for experiment was large, I had tried nearly every drug or combination of drugs that had ever been proposed for the cure or alleviation of this disagreeable malady. Repeated failures and humiliating disappointments had so shaken my faith in the power of drugs over this disease, that I began to use the bromides with a good deal of doubt and hesitation. Greatly to my surprise and gratification, however, I found that I was able to entirely prevent or greatly to alleviate the disease, and have not one single failure to record. The following is the combination I most frequently employ:

℞ Sodii bromidi........................ ʒiv,

Ammonii bromidi........................ ʒij,

Aquæ menthæ piperitæ........................ ℥iij.

M. S. A teaspoonful before meals and at bedtime; begin treatment three days before going on board.

"When preparatory treatment had been neglected and the disease fully established, I put a teaspoonful of the above in a half-tumbler of water, add a drop of ext. ipecac. fluid, and gave a teaspoonful every five minutes; it generally relieves the patient in less than an hour. I have received several letters (guinea enclosed) from passengers asking me to send them the above formula. Next to the bromides, I have found hyoscyamia the most successful remedy. Atropia will frequently afford relief, but is not altogether safe, as I have noticed a few cases of retention of urine to follow its use. I gave nitrate of amyl a fair trial, but it proved a complete failure. I have notes of several cases where the bromides entirely prevented sea-sickness during voyages of from twenty to thirty days, although these patients were always sick on previous voyages."—*Ex.*

**Sulphide of Calcium in Strumous Ophthalmia.**—The good effects resulting from the use of sulphide of calcium in the sores of scrofulous children, and in other affections associated with this diathesis, have been particularly insisted upon by Ringer. It is now my purpose, however, to speak of its value in "strumous ophthalmia." Under this head I allude to phlyctenular and pustular conjunctivitis and keratitis, the characteristics of which I need not further mention. In many of these cases, when other remedies have been used for some time with little or no benefit, the sulphide of calcium has proven of great service. I do not know if it has been much, if at all, recommended in this class of cases, but I was led in the outset to employ it by noticing its beneficial effects in other scrofulous affections, and this especially in the practice of my friend, Dr. Dyson.

The sulphide will be found particularly serviceable in those cases of children with manifest strumous habit, enlarged cervical glands, swollen face, the eyelids tightly closed, photophobia, and where, on opening the eyes, a gush of hot tears is emitted, and examination of the ocular surface discloses one or more phlyctenules on the cornea, or it may be merely increased vascularity of conjunctiva. These cases treated by the ordinary constitutional and local remedies are often tedious, but with the sulphide of calcium, coupled with the usual applications to the eyes, such as atropine and warm fomentations of poppy, or what not, frequently quickly yield a happy result. In other cases also of phlyctenular conjuctivitis or keratitis, and not alone in children, the good effects of this medicine are conspicuous. Of course, like all other drugs, it will be hardly likely to be suitable for, or to benefit, all cases, but I have now employed it with good results so frequently that I am quite satisfied as to its being a useful remedy. After little or no benefit with steel in its various forms, and cod liver oil, the rapid recovery often after the substitution of the sulphide has been astonishing. The mode of administration is generally in the form of a powder, and from gr. 1-10 to gr. ¼ of the sulphide, with a few grains of sugar of milk, are given about three times daily. In this way children take it readily.—Simon Snell, in The Practitioner.—*Mich. News.*

**Iodide of Potassium in Frontal Headache.**—Dr. Haley states, in the Australian Medical Journal for August, that for some years past he has fonnd minimum doses of iodide of potassium of great service in frontal headache. A heavy dull headache situated over the brow, and accompanied by languor, chilliness, and a feeling of general discomfort, with distaste for food, which sometimes approach to nausea, can be completely removed by a two-grain dose dissolved in half a wineglass of water, and this quietly sipped, the whole quantity being taken in about ten minutes. In many cases the effect of these small doses has been simply wonderful. A person who a quarter of an hour before was feeling most miserable, and refused all food, wishing only for quietness, would now take a good meal and resume his wonted cheerfulness. The rapidity with which the iodide acts in these cases constitute its great advantage.—*Louisville Med. News.*

**Treatment of Wine-marks by Electrolysis.**—The object, as in scarification and puncture, is to excite sufficient inflammation to destroy the fine net-work of blood-vessels. A simple needle, or an instrument containing a dozen or more needles, with points upon the same plane and about two millimeters apart, is attached to the negative cord pressed into the skin and the electrolytic action serves to destroy the capillary net-work. The instrument used is a small brass disc, which carries numerous fine cambric needles. When the circuit is completed, a blanching of the tissue for a small space around the needles is immediately observed. With ten or twelve cells of an ordinary zinc and carbon battery, the needles should be allowed to remain ten to thirty seconds, depending upon the delicacy of the skin and the effect produced. The blanching disappears in a few moments. The effect of the electrolysis becomes evident in about three weeks. In aggravated cases, there might be a return of the color, when a very fine and flexible steel needle, introduced in an oblique direction beneath the skin to the depth of a centimetre or more, should be used. By this means he destroyed the larger vessels from which the supply of the capillary vessels was received. The objectionable features were that the operation was a somewhat tedious and painful one, a slight danger of causing suppuration and superficial sloughs, and a tendency to the formation of small keloidal-appearing outgrowths, and sometimes small ulcers and depressed scars, or small, firm vascular nodules. The operation does not leave a perfectly normal skin, but the condition may be greatly improved.

Dr. Sherwell, of Brooklyn, thought that the same results could be obtained by multiple puncture with a needle or disc of needles, the ends of which had been tipped with a caustic, as chromic or carbolic acid.

Dr. Knapp, of New York, suggested that if the starting-point of these marks could be found and destroyed, the remainder would be obliterated. An illustrative case was cited.

Dr. Sherman, of Ogdensburg, cited a case in which spontaneous ulceration occurred at the point, and produced the same result mentioned by Dr. Knapp.—*New York Medical Society-Record.*

**Questions on Vccination.**—Referring to the discussion on vaccination in your journal—

1. I know that some persons are temporarily insusceptible to the effects of vaccine virus. One case, among many in my practice, is this: I was called to a man whom I found with the symptoms of malignant small-pox. I asked him why he had not attended to vaccination? He noticed the tone of fault-finding in my voice, and said: "You cannot blame me, Doctor, for I have been vaccinated twenty-two times, and you have vaccinated me three times yourself." He died in three or four days. There can be no doubt that *he had been insusceptible* to the effects of vaccination; but I have no doubt that if he had been vaccinated within a short period before he was exposed to small-pox, the vaccination would have taken effect and his life would have been saved.

My record of 26,399 vaccinations, to date, show many scores, I think hundreds, of cases, where children were vaccinated with perfect success, who had been unsuccessfully vaccinated previously, from one to fifteen times. It is probable that a considerable number of these had been temporarily insusceptible to the effects of vaccination, though in the greater portion of the cases the failure was the fault of the vaccination.

2. Another case may have some bearing upon another point discussed by your correspondent:

A child was born of a woman sick with modified small-pox or varioloid. The case was severe, the eruption was profuse, and the child was born on the fourth day of the eruption. I vaccinated the child when it was just twenty-four hours old, and at the same time another child about two years old, who had never been vaccinated. The vaccination produced a perfectly typical result in both children; they both remained in the room with their mother, and in the adjoining room, and neither of them had the slightest symptoms of small-pox. The tenement had only two rooms. The disease in the mother had not protected the child from the effects of vaccination. Want of time prevents me from inflicting a much longer story of experience upon you.—E. M. S., *in Med. and Surg. Rep.*

**Treatment of Laryngeal Phthisis.**—Dr. Maurice Schmidt, Frankfort-on-Maine, in Edinburgh Medical Journal, says;

Up to the present time the treatment has almost always been limited to relieving the sufferings of patients as much as possible.

The treatment which I prescribe contains nothing new, the free scarifications excepted. All the other remedies have already been employed by other physiciens. The treatment is divided into two parts—the treatment of the lungs and that of the larynx. As to the first, this is not the place to discuss it. My experience has shown me the superiority of non-medicinal treatmentt physiological if you like.

I would like to say, in a few words, what this treatment is which I make my patients carry out.

(1) Constant residence in an atmosphere as pure as possible both day and night. I induce my patients to keep their windows open during summer or half-closed during winter. I send those who have the means into the mountains, the forests, or to the country. I only make one exception, for patients attacked with inflammation of the larynx, however slight, with whom the climate of the high Alps does not appear to me to agree.

(2) Exercise of the lungs, suited to the strength of each patient to facilitate the expulsion of mucus, and to induce the patients to profit by what remains of their lungs to promote hematosis.

(3) To stimulate the functions of the skin by dry rubbings or by bathing in cold water. A secondary benefit of this remedy is that the patients are hardened against changes of temperature.

(4) A good mixed nourishment, not exclusively animal. During summer, milk cure; during winter, large doses of cod-liver oil, if the patients can bear it, if not they are replaced with food ot a fatty nature. Treatment of affections of the stomach.

**Carcinoma of the Breast.**—Dr. Gross, in a paper read before the New York Academy of Medicine (Boston Medical Journal) draws the following conclusions :

1. That surgical intervention in carcinoma of the breast tends to retard the progress of the disease by preventing local dissemination, implication of associated lymphatic glands, and the development of visceral tumors.

2. That local reproductions do not militate against permanent recovery, provided they are thoroughly and early excised, as soon as they appear; and that lymphatic involvement does not forbid operation, since, in fact, glands were removed in more than one-third of the examples of final cure.

3. That the subjects are almost without exception saved from local and general reproduction, if three years have elapsed after the last operation.

4. That the risk from operations is outweighed by benefits which accrue from them, since they not only add twelve months to the life of the patient, but also cure one-half as many patients as they destroy.

5. That all carcinomas of the breast—if there is no evidence ot metastatic tumors, and if thorough removal is practicable—should be dealt with as early as possible, by amputating the entire mamma, integument and all, dissecting away all the subjacent fascia, opening the axilla, with the view to exploration and removal of all the glands not palpable prior to interference.

**Acute Rheumatism.**—Prof. Latham (Cambridge Medical Society) has advanced a new theory as to the pathological changes in the blood in acute rheumatism, and maintains that the first step was a lowering of the action of the "inhibitory chemical centre," or nervous centre, which controls oxidation in the muscular tissue. Following upon this, the oxygen from the oxyhæmo-globin, instead of entering the muscular tissue to be exhaled therefrom in the form of carbonic acid gas, had its sojourn in the tissue shortened, and passed into the blood in the form of lactic acid (a substance which appears in muscle almost instantaneously with its death). That the oxygen acted also more energetically on the muscular tissue, and the resulting lactic acid being oxidized rapidly in the blood, instead of the muscular tissue, an abnormal amount of heat or pyrexia was developed.

He then argued that quinia lowered temperature by simply impeding the carrying of ozone from the lungs to the tissues by the red corpuscles, as in Binz's experiments with ozonized turpentine and guaiacum; and so the remedy might act beneficially in rheumatism, but would have no effect on the materies morbi.

Salycilic acid, on the other hand, lowered the temperature and cured the disease by mechanically combining with the substances from which the lactic acid is derived, and producing less heat than would from the oxidation of that substance. He recommends large doses to be continued for a long time.—*Dr. Faison, in Transactions of N. C. Med. Society.*

**On the Removal of Warts.**—Dr. W. Allan Jamieson says, in the Practitioner for September, 1881, that chromic acid, one to one of water, is by far the best remedy. The skin round each wart is first protected by painting it with oil, and then the wart itself is soaked with the solution of chromic acid; this absorbs water from the tissues, coagulating and hardening the albuminous tissues at the same time, and the unsightly wart soon disappears. These warts seldom appear after puberty on the hands, but a healthy girl well grown, aged fifteen, came to the writer sometime since with a dozen of them on her hands, which annoyed her for six years. Of course they much interfered with work, being always in the way. Steady use of the chromic acid removed them in a few weeks.—*Independent Practitioner.*

**Vaccination in Scotland.**—Scotland is greatly in favor of vaccination. Dr. Robertson's report of the vaccination of children born in Scotland in 1879, states that only one individual refused to have his children vaccinated. Small-pox has not caused much trouble since 1847, in which year there were 1,246 deaths. In 1880 there were only 10 fatal cases.—*British Medical Journal.*

**Oil of Wintergreen as an Effective Salicylate in Rheumatism.**—An able chemist, namely, Mr. P. Casamajor, of Brooklyn, informs the writer of this paragraph, that arguing from a purely chemical position he expected to obtain better results in acute or subacute rheumatism, and perhaps in chronic rheumatism also, from the use of oil of gaultheria, or wintergreen. This oil is mainly methyl salicylate, and was among the earliest sources of salicylic acid. Mr. Casamajor supposes that this salt of salicylic acid would be easily appropriated in the economy, and would prove more effective than other salicylates of more fixed character. Carrying out his ideas he had treated himself and several friends who had been subjected to rather sharp attacks of rheumatism with oil of wintergreen and with somewhat marked benefit in every case tried. He takes, and gives the oil in doses of ten drops dropped on sugar, and the sugar then mixed with a little water and swallowed about every two hours until the pain is relieved. This simple procedure is well worthy of extended trial and closer observation.—*Dr. Squibb.*

**A New Cinchona Alkaloid, from Cuprea Bark.**—The London Pharmaceutical Journal and Transactions of December 17th, p. 497, has two articles upon a new alkaloid found in the recent coppery colored cinchona bark which has come in large quantities from South America, and is much used by makers of salts of quinia. One paper is by Messrs. B. H. Paul and A. J. Cownley, and the other is by Mr. W. George Whiffen.

These papers concur in their description of an alkaloid not before known, but which is very much like quinia. The sulphate is very much like the sulphate of quinia, but some of the other salts resemble those of cinchonidia. Its chief characteristic as stated by Mr. Whiffen, is its action on polarized light. It rotates the polarized ray to the left more powerfully than quinia, or than any of the alkaloids of cinchona, and therefore he proposes to distinguish it as "ultra-quinine." It has not been separated in any considerable quantity as yet, and nothing seems to be known of its therapeutic value. From its apparent position in the scale of the cinchona alkaloids, it should not be inferior to quinia.—*Dr. Squibb.*

**"Hazeline" as a Local Application in Irritable and Inflamed Piles.**—During the past few months I have given a somewhat extended trial to the new extract of Hamamelis Virginica, called "hazeline." As a local application in irritable and inflamed piles situated at the margin of the anus, where the remedy can be readily applied, I have never met with its equal. In most of the cases submitted to the treatment, the relief was immediate and permanent. My plan has been to have the part bathed in the solution three or four times a day, and a piece of lint dipped in it kept applied to the anus during the intervals. All urgent symptoms have passed away, as a rule, in from twelve to twenty-four hours.—*Braithwaite's Retrospect.*

